# Bile Duct Injury in Children: Is There a Role for Early Endoscopic Retrograde Cholangiopancreatography?

**DOI:** 10.1055/s-0038-1665550

**Published:** 2018-07-12

**Authors:** Akram H. Aljahdali, James J. Murphy

**Affiliations:** 1 Department of Surgery Johns Hopkins Aramco Healthcare Center, Dhahran, Saudi Arabia; 2 Division of Pediatric Surgery, Department of Surgery, University of Vermont Medical Center, Burlington, Vermont

**Keywords:** case series, blunt abdominal trauma, bile leak, ERCP, liver laceration, pediatric trauma

## Abstract

**Introduction** Liver injury is common among pediatric abdominal trauma. Nonoperative management is the standard of care in isolated stable liver injuries. Bile leak is not an uncommon complication in moderate- and high-grade injuries.

**Case series** Three pediatric patients (age: 10–15 years) suffered grade IV liver injuries secondary to blunt abdominal trauma. All developed significant bile leak treated nonoperatively with endoscopic retrograde cholangiopancreatography (ERCP), and patients 1 and 2 were treated with bile duct stent alone. Patient 3 required laparotomy for bile peritonitis and abdominal compartment syndrome followed by interval ERCP and bile duct stent.

**Conclusion** Traumatic bile leaks if not recognized and managed early can result in significant morbidity. This paper describes the presentation and treatment of three pediatric patients with blunt liver trauma complicated by significant bile leaks that were managed successfully with ERCP and bile duct stent. This paper demonstrates the importance of early detection of bile leak to prevent bile peritonitis. Abdominal imaging 4 to 5 days postinjury can help in detecting bile accumulation. We believe that ERCP and bile duct stent are becoming the standard of care in diagnosing and treating traumatic bile leak. This paper confirms the safety and feasibility of this technique in the pediatric population.

The liver is one of the most commonly injured organs in pediatric blunt abdominal trauma. Nonoperative management of blunt hepatic trauma in hemodynamically stable patients has become the standard of care. 1 Bile leak after hepatic injury is a rare complication, but when it does occur, it results in significant morbidities and may prolong hospital stay. Bile leak is usually associated with high-grade liver injuries. 2 The incidence of bile leak post blunt hepatic trauma in children has not yet been defined. In the adult literature, the reported incidence is between 0.5 and 20%. Croce et al reported an incidence of 20% among 70 patients with stable blunt hepatic trauma diagnosed by a hepatoiminodiacetic acid scan (HIDA). 1 Most of these patients had clinically insignificant bile leaks, with the exception of one who was patient treated with percutaneous external drainage. Traditionally, surgical exploration for clinically significant bile leak was recommended. Surgical options include partial hepaticoenterostomy, primary repair of the duct with t-tube insertion, and hepaticoenterostomy. These procedures can be challenging and carry significant morbidities and mortalities.

In the adult population, the use of endoscopic retrograde cholangiopancreatography (ERCP) is a well-established modality for the diagnosis and treatment of postsurgical bile leak and has a high success rate. 3 This technique has been reported in the adult trauma literature, but there is scant evidence for its use in the pediatric population. 4 A few case series in adults have reported successful application of this technique in posttraumatic bile leaks. A few case reports and case series have reported this treatment modality in the pediatric population. Here we report three cases of high-grade liver injuries complicated with clinically significant bile leaks that were successfully treated with this approach.

## Case 1

**Fig. 1:**
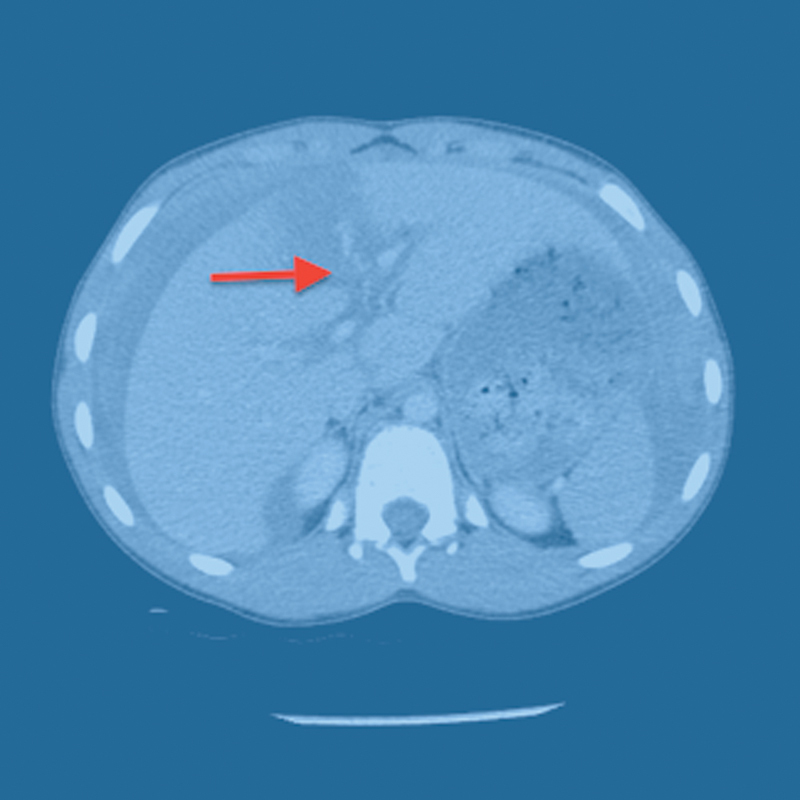
CT (computed tomography) scan of the abdomen of case I showing grade IV liver laceration with free peritoneal fluids. *Arrow* indicates the site of liver laceration.

**Fig. 2:**
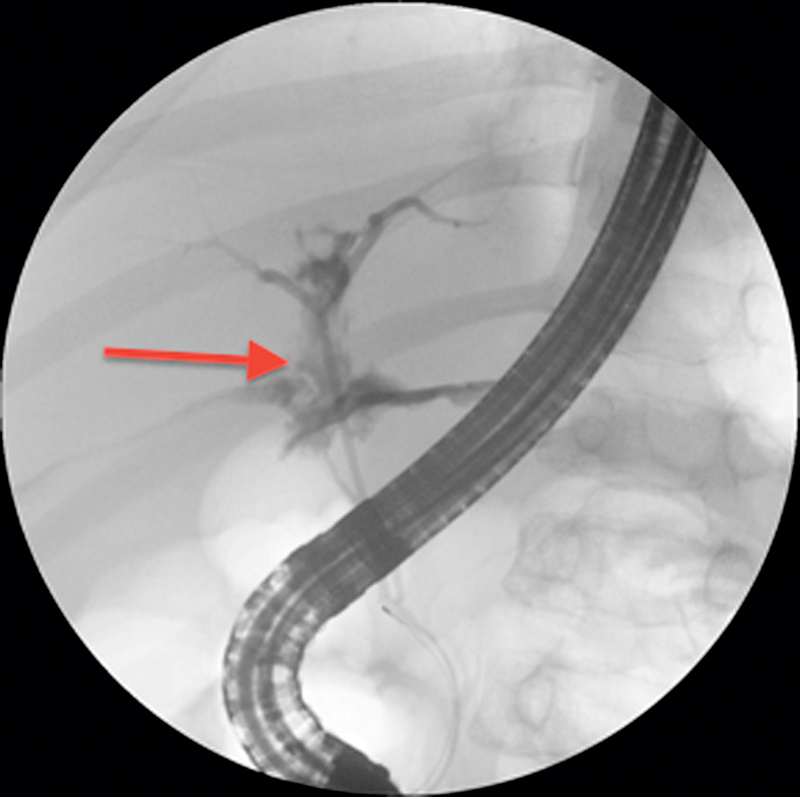
Case I. Contrast leak during endoscopic retrograde cholangiopancreatography from the segmental branches of the right hepatic bile duct. *Arrow* indicates the contrast leakage site within the liver parenchyma.

A 15-year-old male sustained a grade IV liver laceration ( Fig. 1 ) during an all-terrain vehicle accident. He was hemodynamically stable and treated nonoperatively with intensive care unit (ICU) observation for 24 hours and bed rest for a total of 5 days. On hospital day 6, he developed abdominal distension and a high-grade fever. Chest radiograph showed bilateral atelectasis and a right-sided pleural effusion. Abdominal ultrasound (US) revealed a small collection on the right lobe of the liver within the hematoma. Broad-spectrum antibiotics were started, and US-guided drainage of the pleural fluid was performed. The fluid was serous without evidence of infection or bile. The patient developed a worsening ileus and abdominal tenderness a few days later. Repeat abdominal US showed an increase in the amount of free fluid in the right upper quadrant. In addition, his serum conjugated bilirubin was elevated; therefore, suspicion of an ongoing bile leak was high. On hospital day 10 after injury, the patient underwent ERCP ( Fig. 2 ), which demonstrated a leak from one of the secondary bile ducts in the right lobe. Sphincterotomy was performed with insertion of a transampullary stent. Two days later, his ileus resolved. He was started on oral diet, antibiotics were stopped, and then he was discharged home 5 days following the ERCP and stenting. At his 6-week follow-up, the patient had resumed normal physical activity, his bilirubin had normalized, and repeat abdominal US showed complete healing of the liver injury. Biliary stent was removed 8 weeks after placement.

## Case 2

**Fig. 3:**
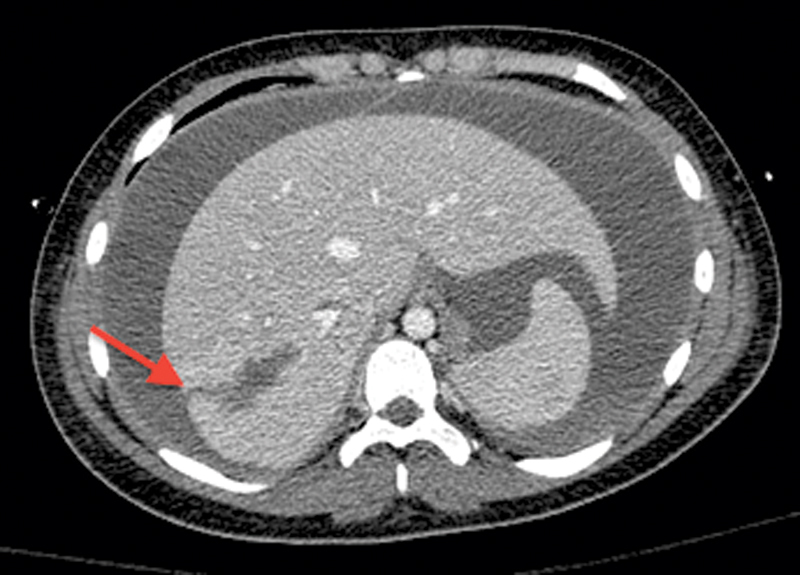
Case 2. CT (computed tomography) scan of the abdomen showing a large amount of free fluids in the abdomen. *Arrow* indicates the site of deep liver laceration.

**Fig. 4:**
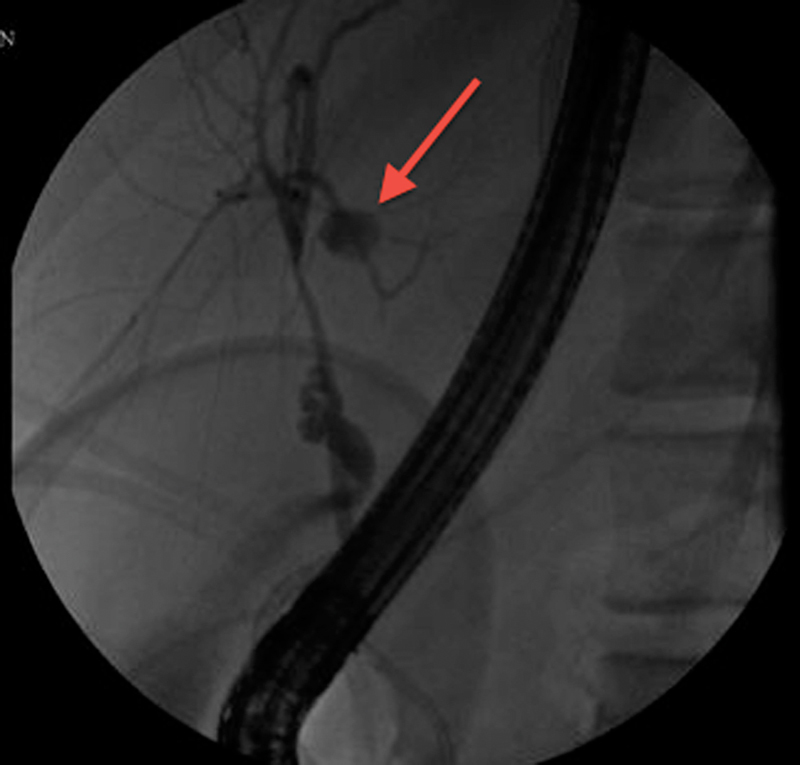
Case 2. Endoscopic retrograde cholangiopancreatography showing bile leak from the segmental branches of the left hepatic bile duct. *Arrow* indicates the site of the bile leak.

A 12-year-old female pedestrian suffered multisystem blunt trauma on the right side of her abdomen after being struck by a sports utility vehicle. The patient suffered a closed head injury, bilateral pulmonary contusions, and grade IV liver laceration. Because the patient was vitally stable, she was observed in the ICU. Seven days after the initial injury, the patient's abdomen became markedly distended and caused respiratory compromise requiring reintubation. Repeat computed tomography (CT) scan of the abdomen ( Fig. 3 ) showed a significant amount of free fluid in the peritoneal cavity. An abdominal drain was inserted under US guidance, and several liters of bloody fluid and bile were removed. Despite the drain, the patient went on to develop infected bile peritonitis (peritoneal culture grew pseudomonas aeruginosa) showing signs of severe sepsis requiring laparotomy and washout, as well as extensive abdominal drainage. Ten days after the laparotomy and 4 weeks after the initial injury, bile leak was persistent and the patient underwent ERCP ( Fig. 4 ), which demonstrated bile leakage from a left second biliary radicle. A transampullary stent was placed. Shortly thereafter, she improved dramatically and was discharged home after a 7-week hospital stay. Bilirubin had returned to normal prior to discharge. A follow-up US 6 weeks later showed no fluid collection or vascular abnormality. The stent was removed 8 weeks later. At her 12-month follow-up, the patient had resumed her normal activity with no residual symptoms.

## Case 3

A 10-year-old boy sustained a handlebar injury while riding a snowmobile. He suffered a grade IV liver laceration with a vascular injury to segment 4 that necessitated emergency angiogram and embolization. Postembolization he developed respiratory distress secondary to increasing abdominal distention. On postinjury day 5, a drain was inserted into the peritoneal cavity which drained approximately 1.5 L of bile. This resulted in clinical improvement, but an HIDA scan postinjury day 8 demonstrated ongoing bile leak from the laceration site. And ERCP demonstrated a leak just distal to the bifurcation of the right hepatic duct. This was treated with stenting and sphincterotomy on postinjury day 10. Unfortunately, the patient continued to have abdominal distention and discomfort and on postinjury day 12, he started deteriorating clinically necessitating an urgent laparotomy and drainage of multiple infected bile and blood collections. Postoperatively he continued to improve slowly until discharge home with close follow-up.

His biliary stent was removed 3 months after insertion. At his 6-month follow-up, he was doing well and had resumed all of his physical activities.

## Discussion

Nonoperative management of liver trauma is the standard of care in children. It is clear that higher grade liver lacerations are associated with higher complication rates. Giss et al reported a 3.8% risk of bile leak and biliary duct related complication among 185 pediatric patients with blunt liver trauma. Those complications were found in grade III and IV injuries only. 5 Free bile leak in the peritoneal cavity can be localized, resulting in biloma formation or diffuse causing bile peritonitis. Clinical diagnosis of bile leak and bile peritonitis can be challenging. Symptoms manifest a few days to weeks after initial injury and are often insidious. Abdominal distension, pain, hyperbilirubinemia, ascites, jaundice, and ileus are the usual symptoms. If the diagnosis is delayed, these patients may develop severe complications such as sepsis, respiratory failure, and abdominal compartment syndrome. This results in serious morbidities and prolonged hospital stay. Several diagnostic tools have been described in the literature such as US, hepatobiliary scintigraphy, magnetic resonance cholangiopancreatography, ERCP, and intraoperative cholangiogram. Church et al reported the case of a 12-year-old boy with high-grade blunt liver laceration complicated by bile leak. 6 Hepatobiliary scintigraphy scan was performed at day 4 followed by washout and intraoperative ERCP and stent. Almaramhi and Al-Qahtani reported five pediatric cases with blunt traumatic bile. They reported that the average time elapse before diagnosis was 24 days. All patients underwent a hepatobiliary scintigraphy scan demonstrating bile leak. Three of the five patients had an extrahepatic bile duct leak and were treated with percutaneous drainage (PD), ERCP, and transampullary stent. The other two had intrahepatic bile duct leak and were treated with PD alone. 7 Griffen et al reported a blunt traumatic bile leak in a 14-year-old boy diagnosed 11 days after admission with paracentesis of large bilious ascites. He was treated with laparoscopic washout, drainage, and ERCP with temporary stent. 8 Castagnetti et al reported five pediatric cases of blunt liver trauma complicated by bile leak. Time elapsed to diagnosis was 3 days to 4 weeks. All patients had at least one ERCP and temporary biliary stent (one patient developed bile duct stricture and septic cholangitis treated successfully with repeat ERCP and dilation of bile duct). 9 Sharpe et al reported the case of an 11-year-old boy with grade II blunt liver injury. Bile leak was diagnosed on postadmission day 16 by hepatobiliary scintigraphy. He was successfully treated with ERCP and transampullary bile duct stent. 10

Early recognition of bile leak is the key to minimizing the risk of complications, shortening hospital stay, and potentially improving the overall morbidity and mortality. Sharif et al reported that the average time lapse to diagnosis of bile leak was 18 days. High-risk patients should have repeat liver function and transaminases checked 2 to 3 days after injury. Total bilirubin level, although sensitive, it not a specific for bile leak. Biliary scintigraphy scan was commonly used among most of the previous case reports and series. It is a very sensitive and safe test to perform. Sharif et al proposed usage of hepatobiliary scintigraphy as a screening tool for bile leak in high-risk patient 2 to 3 days after injury. 11 The drawback of this approach is in its inability to detect many clinically insignificant bile leaks that may resolve spontaneously. 1 An invasive procedure, ERCP carries significant morbidity. Cheng et al reported a 9.7% complication rate from ERCP among 245 pediatric patients, most of these being pancreatitis. 12 Traumatic bile duct injuries following blunt trauma can be associated with pancreatic duct injuries; ERCP serves as diagnostic and definitive therapeutic modality for both. 13 14

This case series demonstrates the importance of early diagnosis of bile leak following high-grade liver injuries. We found that elevated serum bilirubin, worsening abdominal pain, and distension should alert the treating team of the possibility of bile leak. Also, a US of the abdomen can help by demonstrating fluid collection. If the bile collection using laparoscopic exploration and lavage early is recommended to prevent diffuse bile peritonitis as seen in case 2, otherwise inserting an external drain is sufficient for localized collection. HIDA scan can assist in confirming the diagnosis by demonstrating bile leak. If suspicion is high, ERCP can be both diagnostic and therapeutic at the same time. The duration needed for bile duct stent before removal is still unknown. We recommend keeping the stent in for approximately 6 weeks to ensure healing of the ducts. We acknowledge that the number of cases is limited in this study, but when combined with previous reports, the evidence is very strong in supporting the use of ERCP in in bile leak following pediatric liver trauma. More studies comparing ERCP and HIDA scan are required to determine which is the optimal test for detecting the presence of a bile leak. In addition, further study is needed to ascertain whether sphincterotomy alone is sufficient treatment, or if temporary stenting is also necessary.

## Conclusion

Bile duct injuries are uncommon complications of blunt liver trauma in children. The incidence varies between 0.5 and 20%. The majority of these cases suffered high-grade liver trauma. A high index of suspicion is needed to reach the diagnosis as the symptoms are usually insidious and not specific. An HIDA scan, although a sensitive tool, often overestimates the degree of bile leak that may result in unnecessary interventions. This study confirms the safety and efficacy of ERCP in diagnosing and treating pediatric patients with traumatic bile leak. ERCP and temporary transampullary bile duct stent should be considered as the standard of care in the management of bile leak following liver trauma.
